# TAVI-CT score to evaluate the anatomic risk in patients undergoing transcatheter aortic valve implantation

**DOI:** 10.1038/s41598-022-11788-3

**Published:** 2022-05-09

**Authors:** Nicola Corcione, Alberto Morello, Paolo Ferraro, Michele Cimmino, Michele Albanese, Martino Pepe, Palma Luisa Nestola, Salvatore Giordano, Luca Bardi, Giuseppe Biondi-Zoccai, Arturo Giordano

**Affiliations:** 1 Unità Operativa di Interventistica Cardiovascolare, Pineta Grande Hospital, Castel Volturno, Italy; 2 Unità Operativa di Emodinamica, Santa Lucia Hospital, San Giuseppe Vesuviano, Italy; 3 Division of Cardiology, Department of Emergency and Organ Transplantation, University of Bari, Bari, Italy; 4 Division of Cardiology, Department of Medical and Surgical Sciences, Magna Graecia University, Catanzaro, Italy; 5 Department of Medico-Surgical Sciences and Biotechnologies, Sapienza University of Rome, Latina, Italy; 6 Mediterranea Cardiocentro, Naples, Italy

**Keywords:** Cardiology, Interventional cardiology

## Abstract

Transcatheter aortic valve implantation (TAVI) requires thorough preprocedural planning with non-invasive imaging, including computed tomography (CT). The plethora of details obtained with thoraco-abdominal CT represents a challenge for accurate and synthetic decision-making. We devised and tested a comprehensive score suitable to summarize CT exams when planning TAVI. An original comprehensive scoring system (TAVI-CT score) was devised, including details on cardiac, aortic, iliac and femoral artery features. The score was applied to a prospectively collected series of patients undergoing TAVI at our institution, driving decision making on access and prosthesis choice. Different TAVI-CT score groups were compared in terms of procedural success, acute complications, and early clinical outcomes. We included a total of 200 undergoing TAVI between February 2020 and May 2021, with 74 (37.0%) having a low (0–2) TAVI-CT score, 50 (25.0%) having a moderate (3) TAVI-CT score, and 76 (38.0%) having a high (≥ 4) TAVI-CT score. Male gender was the only non-CT variable significantly associated with the TAVI-CT score (p = 0.001). As expected, access choice differed significantly across TAVI-CT scores (p = 0.009), as was device choice, with Portico more favored and Allegra less favored in the highest TAVI-CT score group (p = 0.036). Acute outcomes were similar in the 3 groups, including device and procedural success rates (respectively p = 0.717 and p = 1). One-month follow-up showed similar rates of death, myocardial infarction, stroke, and bleeding, as well as of a composite safety endpoint (all p > 0.05). However, vascular complications were significantly more common in the highest TAVI-CT score group (p = 0.041). The TAVI-CT score is a simple scoring system that could be routinely applied to CT imaging for TAVI planning, if the present hypothesis-generating findings are confirmed in larger prospective studies.

## Introduction

The burden of cardiovascular disease and of degenerative aortic stenosis in particular continues to expand^1–3^. The introduction of transcatheter aortic valve implantation (TAVI) has significantly changed and expanded management options, such that this treatment is being offered to patients at prohibitive, high or even intermediate surgical risk^2,4,5^.

Preliminary planning based on multidimensional imaging is key to achieve favorable outcomes during the procedure as well as subsequently, with multidetector contrast-enhanced computed tomography (CT) playing a central role^6–9^. The benefits of CT include accurate appraisal of vessel dimensions, angles and calcifications, suitable for decision-making in terms of procedural details as well as TAVI device type and size, on top of overall risk assessment and prediction of complications such as permanent pacemaker implantation or prosthesis-patient mismatch^10–18^. However, CT exams may provide a confounding and overwhelming plethora of parameters and measurements, limiting the eventual informativeness of a CT report, leading to inappropriate decisions and strategies, with several apparently useful features actually proving of limited predictive accuracy^9,19^.

Despite many scores suitable for overall risk prediction in patients with severe aortic stenosis and/or those undergoing TAVI^20^, there is limited guidance on how to synthesize the vast number of measurements generated with CT in patients planned for TAVI^21–23^. Building upon extensive experience, thorough review of the literature, and consensus between high volume operators, we generated pre hoc a scoring system, named TAVI-CT score, capable of summarizing poignantly the main findings stemming from a comprehensive CT test for TAVI planning, applying it consistently for several months.

We hereby aim at appraising the role of the TAVI-CT score to inform on procedural success, early and long-term outcomes, as well as choice of access site.

## Methods

### Design and patients

**Figure 1:**
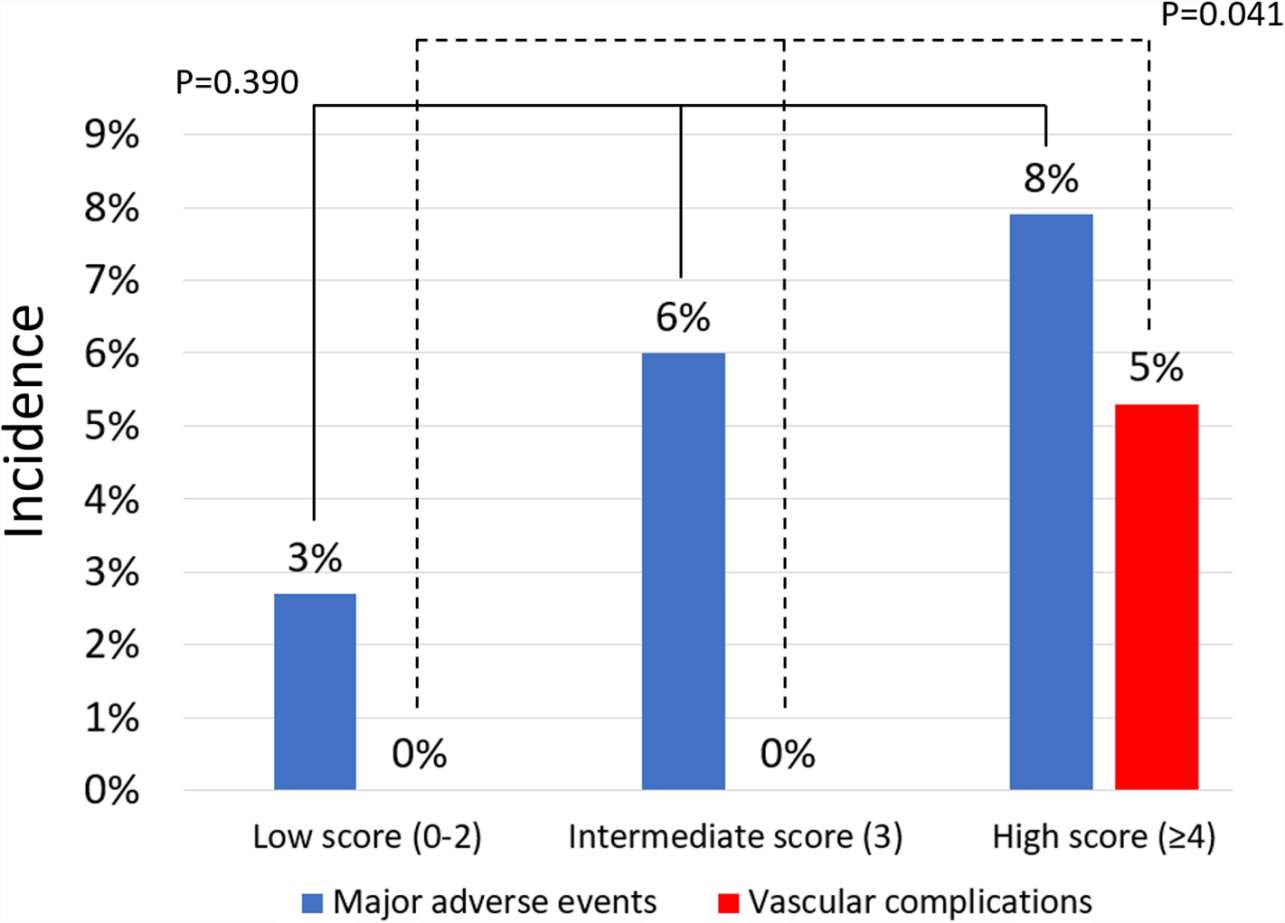
Risk of events according to TAVI-CT (transcatheter aortic valve implantation-computed tomography) score.

This study is a prospective single-center registry using a validated online platform for data collection^24–26^. All methods were performed in accordance with the relevant guidelines and regulations. The study was approved by the Comitato Etico Campania Nord, Caserta, Italy, and all patients provided written informed consent. We included all patients undergoing TAVI for severe aortic stenosis or mixed aortic disease at our institution, which is a large-volume tertiary care center in Southern Italy, specialized in structural heart intervention, with all TAVI performed by two experienced operators (AG, NC), after heart team appraisal. Patients undergoing valve-in-valve TAVI or with missing CT images were excluded (Fig. 1S).

**Figure 2:**
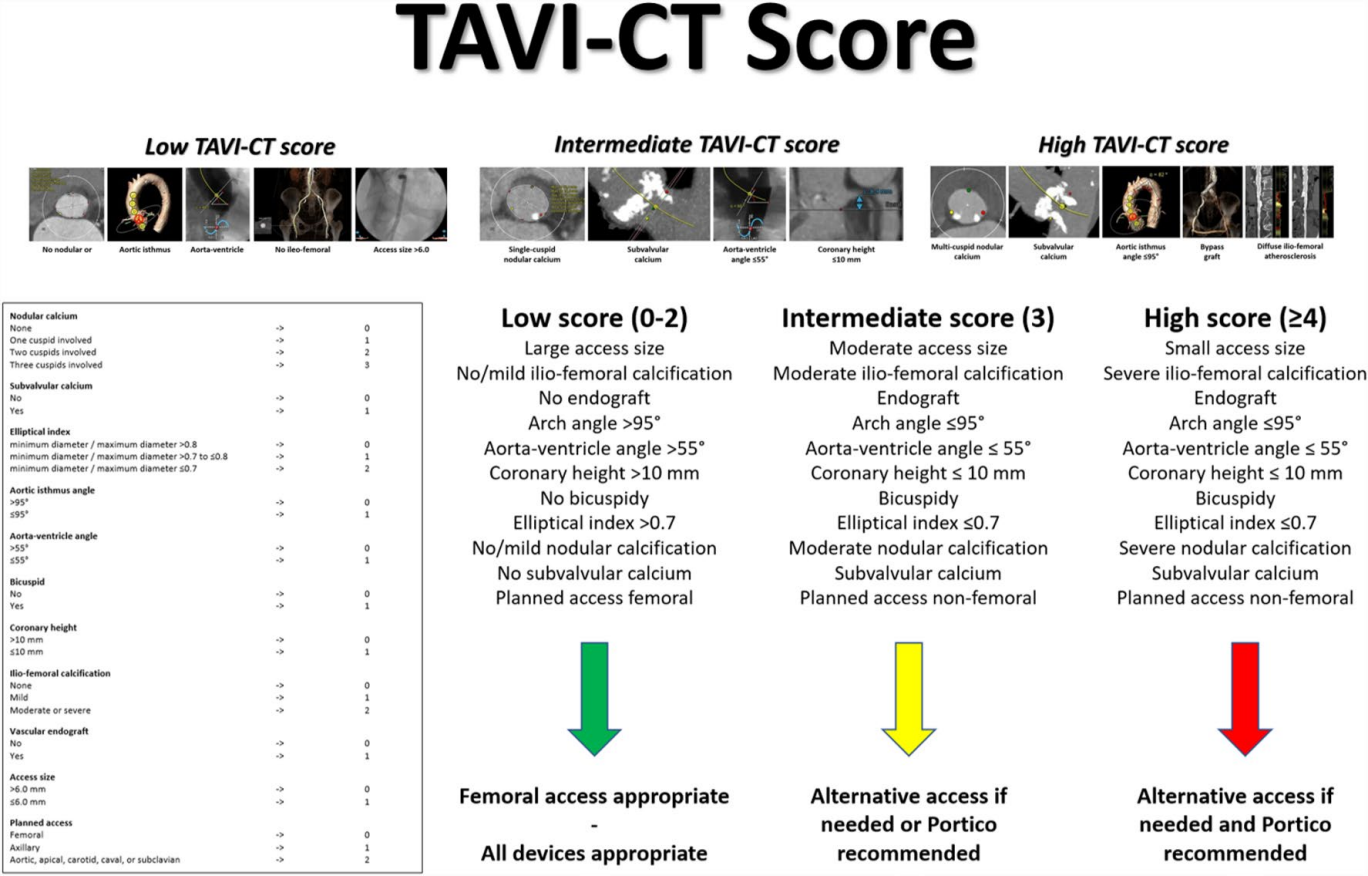
Approach to compute and apply the TAVI-CT (transcatheter aortic valve implantation-computed tomography) score for decision-making.

Before TAVI, all patients were referred for contrast enhanced CT imaging of the chest, abdomen and ilio-femoral axes using 64-row or higher scans, with established methods employed throughout for CT acquisition^16,27,28^. Images were processed offline by a single experience TAVI operator (NC), which had originally devised a summary score, using established methods, and as follows (Fig. 2S)^16,27,28^.

### TAVI-CT score

In particular, nodular calcium was appraised according to Azzalini et al., awarding 3 points in case of involvement of 3 cuspids, 2 points in case of involvement of 2 cuspids, 1 point in case of involvement of 1 cuspid, and 0 points in case of no evidence of nodular calcium^29^. Subvalvular calcium yielded a 1 point score, whereas its absence yielded a 0 point score^16^. The ratio of minimum aortic valve anulus diameter to maximum aortic valve anulus diameter, labelled as elliptical index, was used to generate a 3-tier score, with 2 points yielded in case of an elliptical index ≤ 0.7, 1 point yielded in case of an elliptical index > 0.7 and ≤ 0.8, and 0 points yielded in case of an elliptical index > 0.8^30,31^. One point was yielded in case of an aortic isthmus angle ≤ 95°, with 0 points yielded in case of an aortic isthmus angle > 95°^32,33^. One point was yielded in case of an aorta-ventricle angle > 55°, with 0 points yielded in case of an aorta-ventricle angle ≤ 55°. Bicuspid aortic valve disease using diastolic reconstructions, supplemented by systolic reconstructions when appropriate, according to Alkhadi et al., awarding 1 point in case of bicuspid valve, and 0 points in case of tricuspid valve^34^. Coronary height was measured according to Gooley et al., yielding 1 point in case of height ≤ 10 mm, and 0 points in case of height > 10 mm^35^. Ilio-femoral calcification was appraised according to Okuyama et al., awarding 2 points in case of moderate or severe calcification, 1 point in case of mild calcification, and 0 points in case of no calcification^36^. Access size ≤ 6.0 mm yielded a 1 point, whereas > 6.0 mm yielded 0 points^37^. Finally, planned aortic, apical, carotid, caval or subclavian access yielded 2 points, planned axillary access yielded 1 point, and planned femoral access yielded 0 points.

### Procedures

Procedural planning, including access, approach, predilation, device type and size, postdilation, and ancillary management were all at operators’ discretion, with non-femoral access typically reserved for patients with peripheral artery disease and challenging ilio-femoral anatomy^38^. Similarly, device choice tended to prefer Portico (Abbott Vascular, Santa Clara, CA, USA) devices in cases of challenging aortic valve anatomy.

### Outcomes

Clinical and echocardiographic follow-up, as well as outcome adjudication, was performed in keeping with the Valve Academic Research Consortium (VARC) 3 statement^39^. Specifically, we appraised the 1-month rate of death, cardiac death, stroke, myocardial infarction, bleeding (distinguishing minor, major and disabling), and vascular complication (distinguishing minor and major). In addition, we appraised major adverse events, defined as the composite of death, stroke, myocardial infarction, bleeding, and vascular complication. Notably, events were internally adjudicated by a team of expert clinical researchers, who were not blinded to patient or procedural features.

### Statistical analysis

Continuous variables are reported for descriptive purposes as mean ± standard deviation. Categorical variables are reported accordingly using count (%). For inferential purposes, continuous variables were compared with analysis of variance, whereas categorical variables were compared with Fisher exact test for categorical variables. In addition, areas under the curve (AUC), with 95% confidence intervals, of the receiver-operator characteristic (ROC) curves were computed, providing also accompanying bivariate plots. A complete case analysis approach was used, without missing data imputation. Statistical significance for hypothesis testing was set at the 2-tailed 0.05 level, without multiplicity adjustment. Computations were performed with Stata 13 (StataCorp, College Station, TX, USA).

## Results

A total of 200 patients were enrolled, undergoing TAVI between February 2020 and May 2021 (Tables 1, 2, Fig. 1S). TAVI-CT scores were unevenly distributed, with most patients having a 0–3 score (Table 1S, Fig. 3S). Accordingly, we grouped patients according to different scores as follows: the first group with a 0–2 TAVI-CT score (low TAVI-CT score), the second group with a 3 TAVI-CT score (intermediate TAVI-CT score), and the third group with a TAVI-CT score greater than 3 (high TAVI-CT score). Most baseline features were similar at bivariate analysis according to these 3 groups, except for female gender, which was more prevalent among those with a low TAVI-CT score (p = 0.001). Notably, the most common determinants of a intermediate or high TAVI-CT score were nodular or subvalvular calcium, elliptical annuli, unfavorable angles, ilio-femoral calcification, and small access sizes. The highest scoring patients were a 75-year-old man with an 8 score, and two 75-plus-old men with a 7 score.

**Table 1:** Baseline features according to TAVI-CT (transcatheter aortic valve implantation-computed tomography) score.

Feature	Low score (0–2)	Intermediate score (3)	High score (≥ 4)	p value
Patients	74	50	76	–
Female gender	51 (68.9%)	34 (68.0%)	32 (42.21%)	0.001
Age (years)	81.1 ± 5.4	81.0 ± 6.5	80.4 ± 6.3	0.754
Body mass index (kg/m^2^)	26.9 ± 4.3	27.5 ± 4.4	27.0 ± 4.4	0.748
**Diagnosis**				0.831
Aortic stenosis	38 (51.4%)	23 (46.0%)	38 (50.0%)	
Mixed aortic valve disease	36 (48.7%)	27 (54.0%)	38 (50.0%)	
**Surgical risk**				0.148
Inoperable	1 (1.4%)	0	1 (1.4%)	
High	43 (58.1%)	36 (76.6%)	53 (71.6%)	
Intermediate	30 (40.5%)	11 (23.4%)	20 (27.0%)	
**New York Heart Association class**				0.207
I	1 (1.4%)	0	0	
II	65 (87.4%)	38 (76.0%)	63 (82.9%)	
III	8 (10.8%)	12 (24.0%)	12 (15.8%)	
IV	0	0	1 (1.3%)	
Logistic EuroSCORE	15.3 ± 10.2	17.0 ± 9.5	17.7 ± 13.7	0.444
EuroSCORE II	2.98 ± 2.08	3.46 ± 2.57	3.85 ± 4.76	0.304
Coronary artery disease	7 (9.5%)	7 (14.0%)	14 (18.4%)	0.285
Prior cardiac surgery	5 (6.8%)	7 (14.0%)	9 (11.8%)	0.394
**Prior cerebrovascular event**				0.871
No	69 (93.2%)	44 (88.0%)	69 (90.7%)	
Transient ischemic attack	1 (1.4%)	2 (4.0%)	2 (2.6%)	
Stroke	4 (5.4%)	4 (8.0%)	5 (6.6%)	
Peripheral artery disease	13 (17.6%)	9 (18.0%)	35 (46.1%)	
Estimated glomerular filtration rate (mL/min/1.73 m^2^)	61.7 ± 17.3	59.6 ± 20.1	66.7 ± 21.6	0.114
Chronic obstructive pulmonary disease	18 (24.3%)	16 (32.0%)	25 (32.9%)	0.454

**Table 2:** Imaging features according to TAVI-CT (transcatheter aortic valve implantation-computed tomography) score.

Feature	Low score (0–2)	Intermediate score (3)	High score (≥ 4)	p value
Patients	74	50	76	–
Aortic valve area (cm2)	0.63 ± 0.14	0.61 ± 0.10	0.60 ± 0.13	0.586
Left ventricular ejection fraction (%)	52.2 ± 7.7	51.2 ± 8.4	51.1 ± 8.8	0.693
Mean valve gradient (mm Hg)	49.3 ± 19.2	46.6 ± 19.7	48.3 ± 15.1	0.707
**Aortic regurgitation**				0.417
None	22 (29.7%)	17 (34.0%)	24 (31.6%)	
1+	26 (35.1%)	21 (42.0%)	28 (36.8%)	
2+	20 (27.0%)	5 (10.0%)	16 (21.1%)	
3+	6 (8.1%)	7 (14.0%)	8 (10.5%)	
Porcelain aorta	0	1 (2.0%)	3 (4.0%)	0.296
TAVI-CT score	1.6 ± 0.6	3 ± 0	4.9 ± 1.2	< 0.001
0	3 (4.1%)	0	0	< 0.001
1	23 (31.1%)	0	0	
2	48 (64.9%)	0	0	
3	0	50 (100%)	0	
4	0	0	42 (55.3%)	
5	0	0	14 (18.4%)	
> 5	0	0	20 (26.2%)	
**TAVI-CT score components**				
**Nodular calcium (scored from 0 to 3)**				< 0.001
None	68 (93.2%)	38 (76.0%)	34 (44.7%)	
1 cuspid involved	5 (6.9%)	12 (24.0%)	32 (42.1%)	
2 cuspids involved	0	0	10 (13.2%)	
3 cuspids involved	0	0	0	
Subvalvular calcium (scored from 0 to 1)	2 (2.7%)	4 (8.0%)	26 (34.7%)	< 0.001
**Elliptical index (scored from 0 to 2)**				< 0.001
≤0.7	20 (27.0%)	8 (16.0%)	6 (7.9%)	
>0.7 to ≤0.8	49 (66.2%)	23 (46.0%)	31 (40.8%)	
>0.8	5 (6.8%)	19 (38.0%)	39 (51.3%)	
Aortic isthmus angle ≤ 95° (scored from 0 to 1)	3 (5.0%)	15 (31.9%)	23 (32.4%)	< 0.001
Aorta-ventricle angle ≤ 55° (scored from 0 to 1)	41 (66.1%)	34 (70.8%)	62 (83.8%)	0.051
Bicuspid (scored from 0 to 1)	0	3 (6.1%)	5 (6.6%)	0.049
Coronary height ≤ 10 mm (scored from 0 to 1)	2 (2.7%)	0	10 (13.2%)	0.003
**Ilio-femoral calcification (scored from 0 to 2)**				< 0.001
None	67 (90.5%)	37 (74.0%)	40 (53.3%)	
Mild	7 (9.5%)	11 (22.0%)	21 (28.0%)	
Moderate or severe	0	2 (4.0%)	14 (18.7%)	
Vascular endograft (scored from 0 to 1)	0	1 (2.0%)	5 (6.6%)	0.045
Access size ≤ 6.0 mm (scored from 0 to 1)	0	3 (6.0%)	12 (15.8%)	< 0.001
**Planned access (scored from 0 to 2)**				< 0.001
Femoral	74 (100%)	48 (96.0%)	63 (82.9%)	
Axillary	0	2 (4.0%)	9 (11.8%)	
Aortic, apical, caval, carotid, or subclavian	0	0	4 (5.3%)	

Procedural features were also similar across the 3 groups (Table 3), except for access site, with non-femoral access more common in patients with a high TAVI-CT score (p = 0.009), and device choice, with Portico being relatively more common in the same group of patients (p = 0.036). Irrespectively, acute results were similarly satisfactory in the 3 groups, with device success ranging between 98.0% and 100% (p = 0.717) and procedural success 100% in all groups (p = 1).

**Table 3:** Procedural features according to TAVI-CT (transcatheter aortic valve implantation-computed tomography) score.

Feature	Low score (0–2)	Intermediate score (3)	High score (≥ 4)	p value
Patients	74	50	76	–
**Anesthesia**				0.427
Local	72 (97.3%)	49 (98.0%)	75 (98.7%)	
Spinal	0	1 (2.0%)	0	
General	2 (2.7%)	0	1 (1.3%)	
**Actual access**				0.009
Femoral	74 (100%)	48 (96.0%)	67 (88.2%)	
Axillary	0	2 (4.0%)	7 (9.2%)	
Subclavian	0	0	2 (2.6%)	
Percutaneous approach	74 (100%)	50 (100%)	76 (100%)	1
Predilation	54 (73.0%)	42 (84.0%)	61 (80.3%)	0.326
**Device**				0.036
Allegra	6 (8.1%)	5 (10.0%)	1 (1.3%)	
Evolut Pro/R	28 (37.8%)	10 (20.0%)	23 (30.3%)	
Portico	40 (54.1%)	35 (70.0%)	52 (68.4%)	
Bailout valve-in-valve	0	1 (2.0%)	1 (1.3%)	0.717
Postdilation	37 (50.0%)	31 (62.0%)	48 (63.2%)	0.222
Postdilation balloon diameter (mm)	23.4 ± 2.1	23.9 ± 2.4	23.8 ± 2.0	0.565
Hemostasis with 2 ProGlides	74 (100%)	50 (100%)	76 (100%)	1
Contrast volume (mL)	77.2 ± 18.3	76.9 ± 18.3	79.2 ± 15.3	0.689
Fluoroscopy time (min)	17.2 ± 4.2	17.1 ± 4.1	18.5 ± 7.4	0.296
Procedural time (min)	54.9 ± 8.4	55.2 ± 7.2	56.5 ± 8.5	0.437
Device success	74 (100%)	49 (98.0%)	75 (98.7%)	0.717
Procedural success	74 (100%)	50 (100%)	76 (100%)	1

One-month follow-up confirmed the favorable clinical results obtained acutely and during hospital stay (Table 4), which was not significantly different (p = 0.427). Notably, the rate of major adverse events, while non-significantly different (p = 0.390), appear to increase progressively from the low score group (2.7%) to the intermediate score group (6.0%) and to the high score group (7.9%) (Fig. 1). Indeed, only the rate of vascular complications appeared significantly different in the 3 groups, with no vascular complication in the low or intermediate score groups, and 4 minor vascular complications in the high score group (p = 0.041). Similar findings were obtained when discounting planned access from the computation of the TAVI-CT score (Table 2S).

**Table 4:** Clinical and imaging outcomes at 1-month follow-up according to TAVI-CT (transcatheter aortic valve implantation-computed tomography) score.

Feature	Low score (0–2)	Intermediate score (3)	High score (≥ 4)	p value
Patients	74	50	76	–
Total length of stay (days)	5.4 ± 1.9	5.7 ± 1.8	5.9 ± 2.2	0.329
Major adverse event*	2 (2.7%)	3 (6.0%)	6 (7.9%)	0.390
Death	1 (1.4%)	1 (2.0%)	1 (1.3%)	1
Cardiac death	1 (1.4%)	0	1 (1.3%)	1
Myocardial infarction	1 (1.4%)	1 (2.0%)	0	0.526
Stroke	0	1 (2.0%)	0	0.250
**Bleeding**				0.469
None	74 (100%)	49 (98%)	74 (97.4%)	
Type 1	0	1 (2.0%)	2 (2.6%)	
Type 2	0	0	0	
Type 3	0	0	0	
Type 4	0	0	0	
**Vascular complication**				0.041
None	74 (100%)	50 (100%)	72 (94.7%)	
Minor	0	0	4 (5.3%)	
Major	0	0	0	
Surgical conversion	0	0	0	1
Aortic dissection	0	0	0	1
Anulus rupture	0	0	0	1
Bailout percutaneous coronary intervention	1 (1.4%)	1 (2.0%)	0	0.526
Permanent pacemaker implantation	6 (8.1%)	6 (12.0%)	10 (13.2%)	0.599
Left ventricular ejection fraction (%)	52.7 ± 8.0	52.1 ± 9.3	52.3 ± 8.9	0.902
Peak gradient (mm Hg)	13.9 ± 5.1	13.5 ± 5.6	13.9 ± 6.1	0.888
Mean gradient (mm Hg)	7.9 ± 3.2	7.6 ± 3.5	7.9 ± 3.5	0.830
**Aortic regurgitation**				0.113
None	15 (20.6%)	5 (10.0%)	15 (19.7%)	
1+	55 (75.3%)	44 (88.0%)	53 (69.7%)	
2+	3 (4.1%)	1 (2.0%)	8 (10.5%)	

*Composite of death, myocardial infarction, stroke, bleeding, or vascular complication.

Analysis of diagnostic accuracy confirmed the previous results (Table 3S), showing that the TAVI-CT score could have a limited predictive role for major adverse events (e.g. AUC = 0.66 [0.50–0.83]), whereas the predictive accuracy for vascular complications was substantial, with AUC = 0.88 (0.71–1.00) for TAVI-CT score (Fig. 4S), AUC = 0.90 (0.74–1.00) for TAVI-CT score excluding planned access (Fig. 5S), AUC = 0.63 (0.56–0.70) for the abridged, 3-tiered version of the TAVI-CT score, and AUC = 0.62 (0.48–0.77) for the abridged, 3-tiered version of the TAVI-CT score.

Further proof of the usefulness of the TAVI-CT score is that none of its component, individually, was significantly associated with major adverse events (Table 4S). Conversely, elliptical index, ilio-femoral calcification, and access size ≤ 6.0 mm were all individually and significantly associated with the risk of vascular complications (all p < 0.05, Table 5S).

## Discussion

The success of TAVI continuous momentously, thanks to improvements in patient selection, device evolution, procedural refinements, and ad hoc ancillary medical management^2,4,5,24–26,38^. Indeed, with the ongoing expansion in the indications for TAVI, it is crucial to ensure adequate pre-procedural evaluation and planning are performed, in a logic of tailored access and device choice. Computed tomography offers a wealth of information suitable to guide operators envisioning TAVI, either before heart team discussion, or after the decision for this treatment has been taken^28^. Yet, CT interpretation may be challenging and overwhelming even for expert readers and operators.

While to date efforts at synthesizing the appraisal of pre-TAVI CT have been mainly limited as specific analysis (e.g. valve calcium quantification), there is a paucity of studies aimed at summarizing all features which may impact on operative and post-operative management.

In the present work, we originally aimed at devising, pre hoc, a semiquantitative scoring system suitable to capture all important features and assessments stemming from pre-TAVI CT, labelled TAVI-CT score, ranging from coronary height to ilio-femoral vessels. The score is very easily performed and informative, ranging from 0 to a theoretical maximum of 14. Intriguingly, the score was not associated with baseline features, except for female gender (with women typically having lower scores). Parsimoniously exploiting the score to generate 3 groups, lead to a low TAVI-CT score group (with scores ranging from 0 to 2), an intermediate TAVI-CT score group (with scores of 3), and a high TAVI-CT score group (with scores of 4 or more).

Female gender was associated with lower TAVI-CT scores, despite the typically smaller vessels of these patients. Indeed, this finding is reassuring and confirms the rosy outlook of TAVI even in female patients with severe aortic valve disease at intermediate, high or prohibitive surgical risk. Access and device choice were different in the TAVI-CT score groups, with non-femoral access and Portico more common in patients with intermediate or high scores, as appropriately expected given the need to minimize access site complications and ensure a flexible device was chosen for TAVI. Clinical outcomes were largely similar across the score groups, despite a linear, albeit non-significant, increase in major adverse events, and a significant increase in vascular complications in patients with higher TAVI-CT scores.

The goal of improving the evaluation of patients with indication to TAVI based on pre-procedural CT is meaningful and worthy of pursue. Indeed, other researchers have attempted at capitalizing the diagnostic yield of CT using more readily applicable and sanctionable scores^21,29,40,41^. For instance, the ilio-femoral tortuosity (IFT) score has been recently proposed by Mach et al., and proved to predict a composite of bleeding or access complications^21^. Notably, the TAVI-CT score should not be viewed as an alternative to established operative or prognostic scores, such as the EuroSCORE, the STS score, or, as recently suggested, the CHA_2_DS_2_-VASC score, the HAS-BLED score, or the combined CHADS-BLED score, as well as more novel modeling approaches^20,42–44^. Instead, the TAVI-CT score should be considered as an adjunct tool suitable to simplify pre-procedural evaluation, choice between TAVI and surgical aortic valve replacement, and detailed TAVI planning. Specifically, we may suggest that patients with a low TAVI-CT score could be treated with default femoral access and with any TAVI device (Fig. 2). Instead, in patients with intermediate or high TAVI-CT scores, axillary access could be considered more liberally in case of peripheral artery disease, and more flexible devices such as Portico could be used routinely^24^.

This work has several limitations, including the small sample size, the low event rates, the absence of independent event adjudication by a clinical event committee, and the lack of machine learning analysis to quantify candidate factors for entry and specific weighing in the eventual score. Indeed, the score was devised by an experienced operator pre-hoc, thus representing an expert synthesis of his expertise in evaluating pre-TAVI CT and weighing salient features for TAVI planning. Furthermore, we cannot exclude that decision-making based on expert knowledge led to procedural adjustments eventually mitigating the adverse impact of a specific TAVI-CT feature or a globally increased score. Accordingly, this work represents a pilot study, and multicenter studies are warranted to confirm or disprove the present findings. Indeed, it is plausible that only some of the components of the TAVI-CT score are actually informative for procedural planning or outcomes.

In conclusion, the TAVI-CT score is a simple scoring system that could be routinely applied to CT imaging for TAVI planning, if the present hypothesis-generating findings are confirmed in larger prospective studies.

## Supplementary Information

Supplementary Information.
