# Supplementary material for: TAVI-CT score to evaluate the anatomic risk in patients undergoing transcatheter aortic valve implantation
Source: Sci Rep. 2022 May 9;12:7612. doi: 10.1038/s41598-022-11788-3 (PMC9085825; doi:10.1038/s41598-022-11788-3)
Supplement: Supplementary file 1 — Supplementary Information. [file 41598_2022_11788_MOESM1_ESM.docx]

**TAVI-CT score to evaluate the anatomic risk in patients undergoing transcatheter aortic valve implantation**

**Online supplement**

**Table 1S.** Distribution of TAVI-CT (transcatheter aortic valve implantation-computed tomography) score and its components.

| **Feature** | **Count (%)** |
| --- | --- |
| Patients | 200 |
| TAVI-CT score |  |
| 1 | 23 (11.5%) |
| 2 | 48 (24.0%) |
| 3 | 50 (25.0%) |
| 4 | 42 (21.0%) |
| 5 | 14 (7.0%) |
| 6 | 12 (6.0%) |
| 7 | 5 (2.5%) |
| 8 | 2 (1.0%) |
| 9 | 1 (0.5%) |
| TAVI-CT score components |  |
| Nodular calcium (scored from 0 to 3) |  |
| None | 140 (70.4%) |
| 1 cuspid involved | 49 (24.6%) |
| 2 cuspids involved | 10 (5.0%) |
| 3 cuspids involved | 0 |
| Subvalvular calcium (scored from 0 to 1) | 32 (16.2%) |
| Elliptical index (scored from 0 to 2) |  |
| ≤0.7 | 34 (17.0%) |
| >0.7 to ≤0.8 | 103 (51.5%) |
| >0.8 | 63 (31.5%) |
| Aortic isthmus angle ≤95° (scored from 0 to 1) | 41 (23.0%) |
| Aorta-ventricle angle ≤55° (scored from 0 to 1) | 137 (74.5%) |
| Bicuspid (scored from 0 to 1) | 8 (4.0%) |
| Coronary height ≤10 mm (scored from 0 to 1) | 12 (6.0%) |
| Ilio-femoral calcification (scored from 0 to 2) |  |
| None | 144 (72.4%) |
| Mild | 39 (19.6%) |
| Moderate or severe | 16 (8.0%) |
| Vascular endograft (scored from 0 to 1) | 6 (3.0%) |
| Access size ≤6.0 mm (scored from 0 to 1) | 15 (7.5%) |
| Planned access (scored from 0 to 2) |  |
| Femoral | 185 (92.5%) |
| Axillary | 11 (5.5%) |
| Aortic, apical, caval, carotid, or subclavian | 4 (2.0%) |

**Table 2S.** Clinical and imaging outcomes at 1-month follow-up discounting planned access from the TAVI-CT (transcatheter aortic valve implantation-computed tomography) score.

| **Feature** | **Low score (0-2)** | **Intermediate score (3)** | **High score (≥4)** | **P value** |
| --- | --- | --- | --- | --- |
| Patients | 80 | 57 | 63 | - |
| Total length of stay (days) | 5.4±1.8 | 5.7±1.9 | 5.9±2.2 | 0.363 |
| Major adverse event* | 2 (2.5%) | 5 (8.8%) | 4 (6.4%) | 0.263 |
| Death | 1 (1.3%) | 2 (3.5%) | 0 | 0.374 |
| Cardiac death | 1 (1.3%) | 1 (1.8%) | 0 | 0.747 |
| Myocardial infarction | 1 (1.3%) | 1 (1.8%) | 0 | 0.747 |
| Stroke | 0 | 1 (1.8%) | 0 | 0.285 |
| Bleeding |  |  |  | 0.192 |
| None | 80 (100%) | 55 (96.5%) | 62 (98.4%) |  |
| Type 1 | 0 | 2 (3.5%) | 1 (1.6%) |  |
| Type 2 | 0 | 0 | 0 |  |
| Type 3 | 0 | 0 | 0 |  |
| Type 4 | 0 | 0 | 0 |  |
| Vascular complication |  |  |  | 0.103 |
| None | 80 (100%) | 56 (98.3%) | 60 (95.2%) |  |
| Minor | 0 | 1 (1.8%) | 3 (4.8%) |  |
| Major | 0 | 0 | 0 |  |
| Surgical conversion | 0 | 0 | 0 | 1 |
| Aortic dissection | 0 | 0 | 0 | 1 |
| Anulus rupture | 0 | 0 | 0 | 1 |
| Bailout percutaneous coronary intervention | 1 (1.3%) | 1 (1.8%) | 0 | 0.747 |
| Permanent pacemaker implantation | 6 (7.5%) | 7 (12.3%) | 9 (14.3%) | 0.413 |
| Left ventricular ejection fraction (%) | 52.3±8.0 | 52.3±9.3 | 52.5±9.0 | 0.989 |
| Peak gradient (mm Hg) | 13.6±5.1 | 13.4±5.6 | 14.5±6.3 | 0.531 |
| Mean gradient (mm Hg) | 7.7±3.2 | 7.5±3.3 | 8.2±3.6 | 0.522 |
| Aortic regurgitation |  |  |  | 0.019 |
| None | 18 (22.5%) | 6 (10.5%) | 12 (19.1%) |  |
| 1+ | 57 (71.3%) | 51 (89.5%) | 44 (98.4%) |  |
| 2+ | 5 (6.3%) | 0 | 7 (11.1%) |  |

*composite of death, myocardial infarction, stroke, bleeding, or vascular complication

**Table 3S.** Areas under the curve (AUC) with 95% confidence intervals of the receiver-operator characteristic curves for different versions of the TAVI-CT (transcatheter aortic valve implantation-computed tomography) score.

| **Feature** | **TAVI-CT score** | **TAVI-CT score, excluding planned access** | **Abridged TAVI-CT score** | **Abridged TAVI-CT score, excluding planned access** |
| --- | --- | --- | --- | --- |
| Major adverse event* | 0.66  (0.50-0.83) | 0.66  (0.49-0.83) | 0.63  (0.45-0.81) | 0.60  (0.46-0.74) |
| Vascular complication | 0.88  (0.71-1.00) | 0.90  (0.74-1.00) | 0.63  (0.56-0.70) | 0.62  (0.48-0.77) |

*composite of death, myocardial infarction, stroke, bleeding, or vascular complication

**Table 4S.** Impact of individual components of the TAVI-CT (transcatheter aortic valve implantation-computed tomography) score on the risk of major adverse events.*

| **Feature** | **No major adverse event** | **Major adverse event** | **P value** |
| --- | --- | --- | --- |
| Patients | 189 | 11 | - |
| Nodular calcium (scored from 0 to 3) |  |  | 0.343 |
| None | 131 (69.7%) | 9 (81.8%) |  |
| 1 cuspid involved | 48 (25.5%) | 1 (9.1%) |  |
| 2 cuspids involved | 9 (4.8%) | 1 (9.1%) |  |
| 3 cuspids involved | 0 | 0 |  |
| Subvalvular calcium (scored from 0 to 1) | 32 (17.1%) | 0 | 0.217 |
| Elliptical index (scored from 0 to 2) |  |  | 0.335 |
| ≤0.7 | 33 (17.5%) | 1 (9.1%) |  |
| >0.7 to ≤0.8 | 99 (52.4%) | 4 (36.4%) |  |
| >0.8 | 57 (30.2%) | 6 (54.6%) |  |
| Aortic isthmus angle ≤95° (scored from 0 to 1) | 37 (22.0%) | 4 (40.0%) | 0.242 |
| Aorta-ventricle angle ≤55° (scored from 0 to 1) | 129 (74.1%) | 8 (80.0%) | 1 |
| Bicuspid (scored from 0 to 1) | 7 (3.7%) | 1 (9.1%) | 0.371 |
| Coronary height ≤10 mm (scored from 0 to 1) | 11 (5.8%) | 1 (9.1%) | 0.503 |
| Ilio-femoral calcification (scored from 0 to 2) |  |  | 0.084 |
| None | 137 (72.9%) | 7 (63.6%) |  |
| Mild | 38 (20.2%) | 1 (9.1%) |  |
| Moderate or severe | 13 (6.9%) | 3 (27.3%) |  |
| Vascular endograft (scored from 0 to 1) | 5 (2.7%) | 1 (9.1%) | 0.291 |
| Access size ≤6.0 mm (scored from 0 to 1) | 13 (6.9%) | 2 (18.2%) | 0.195 |
| Planned access (scored from 0 to 2) |  |  | 0.195 |
| Femoral | 176 (93.1%) | 9 (81.8%) |  |
| Axillary | 9 (4.8%) | 2 (18.2%) |  |
| Aortic, apical, caval, carotid, or subclavian | 4 (2.1%) | 0 |  |

*composite of death, myocardial infarction, stroke, bleeding, or vascular complication

**Table 5S.** Impact of individual components of the TAVI-CT (transcatheter aortic valve implantation-computed tomography) score on the risk of vascular complications.*

| **Feature** | **No major adverse event** | **Major adverse event** | **P value** |
| --- | --- | --- | --- |
| Patients | 196 | 4 | - |
| Nodular calcium (scored from 0 to 3) |  |  | 0.156 |
| None | 137 (70.3%) | 3 (75.0%) |  |
| 1 cuspid involved | 49 (25.1%) | 0 |  |
| 2 cuspids involved | 9 (4.6%) | 1 (25.0%) |  |
| 3 cuspids involved |  |  |  |
| Subvalvular calcium (scored from 0 to 1) | 32 (16.5%) | 0 | 1 |
| Elliptical index (scored from 0 to 2) |  |  | 0.016 |
| ≤0.7 | 34 (17.4%) | 0 |  |
| >0.7 to ≤0.8 | 103 (52.6%) | 0 |  |
| >0.8 | 59 (30.1%) | 4 (100%) |  |
| Aortic isthmus angle ≤95° (scored from 0 to 1) | 40 (22.9%) | 1 (33.3%) | 0.546 |
| Aorta-ventricle angle ≤55° (scored from 0 to 1) | 134 (74.0%) | 3 (100%) | 0.571 |
| Bicuspid (scored from 0 to 1) | 7 (3.6%) | 1 (25.0%) | 0.152 |
| Coronary height ≤10 mm (scored from 0 to 1) | 12 (6.1%) | 0 | 1 |
| Ilio-femoral calcification (scored from 0 to 2) |  |  | 0.017 |
| None | 143 (73.3%) | 1 (25.0%) |  |
| Mild | 38 (19.5%) | 1 (25.0%) |  |
| Moderate or severe | 14 (7.2%) | 2 (50.0%) |  |
| Vascular endograft (scored from 0 to 1) | 5 (2.6%) | 1 (25.0%) | 0.116 |
| Access size ≤6.0 mm (scored from 0 to 1) | 13 (6.6%) | 2 (50.0%) | 0.029 |
| Planned access (scored from 0 to 2) |  |  | 0.270 |
| Femoral | 182 (92.9%) | 3 (75.0%) |  |
| Axillary | 10 (5.1%) | 1 (25.0%) |  |
| Aortic, apical, caval, carotid, or subclavian | 4 (2.0%) | 0 |  |

**Figure 1S.** Flowchart detailing patient selection.


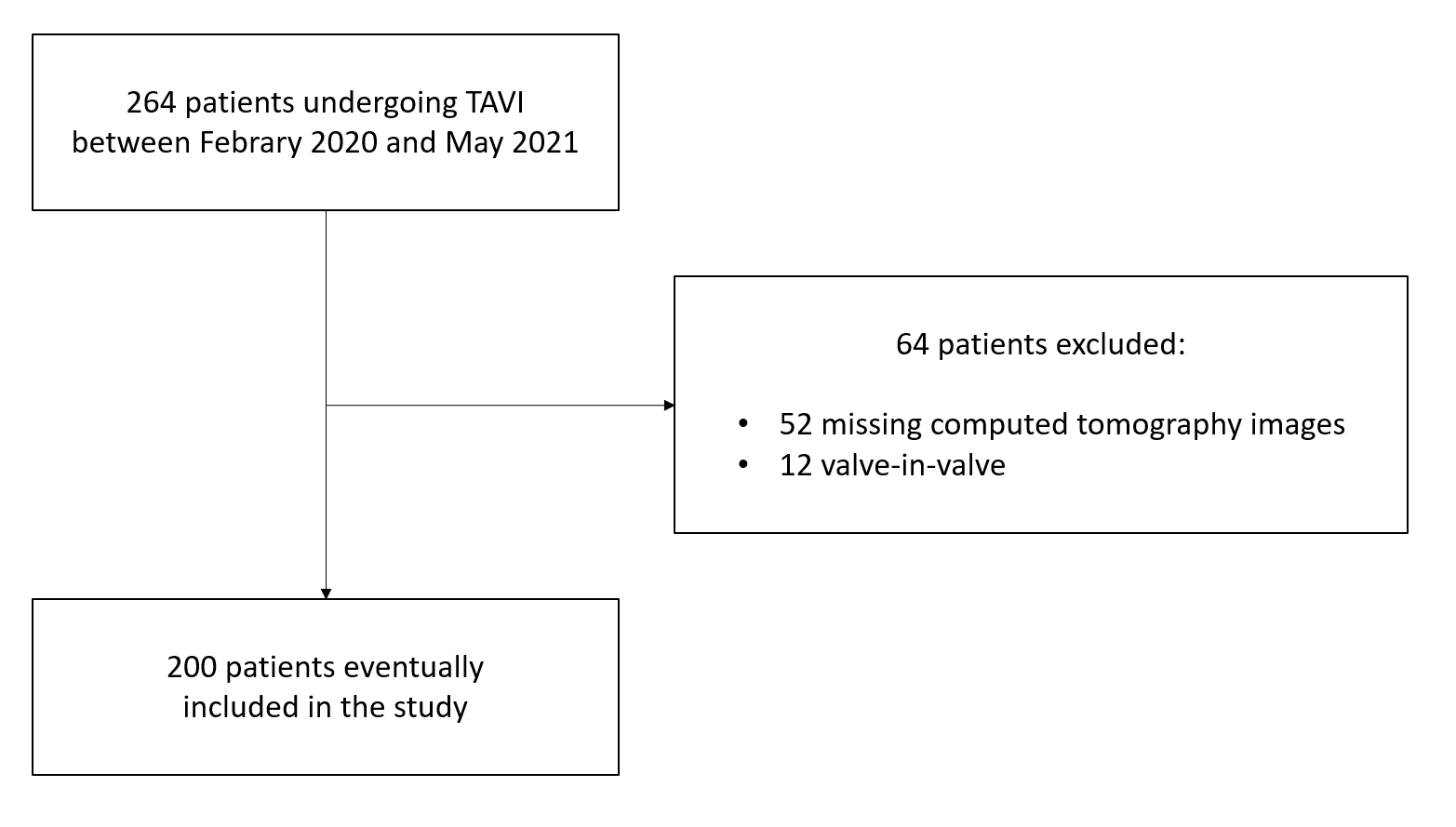


**Figure 2S.** Computation of the TAVI-CT (transcatheter aortic valve implantation-computed tomography) score.

**Nodular calcium**

None -> 0

One cuspid involved -> 1

Two cuspids involved -> 2

Three cuspids involved -> 3

**Subvalvular calcium**

No -> 0

Yes -> 1

**Elliptical index**

minimum diameter / maximum diameter >0.8 -> 0

minimum diameter / maximum diameter >0.7 to ≤0.8 -> 1

minimum diameter / maximum diameter ≤0.7 -> 2

**Aortic isthmus angle**

>95° -> 0

≤95° -> 1

**Aorta-ventricle angle**

>55° -> 0

≤55° -> 1

**Bicuspid**

No -> 0

Yes -> 1

**Coronary height**

>10 mm -> 0

≤10 mm -> 1

**Ilio-femoral calcification**

None -> 0

Mild -> 1

Moderate or severe -> 2

**Vascular endograft**

No -> 0

Yes -> 1

**Access size**

>6.0 mm -> 0

≤6.0 mm -> 1

**Planned access**

Femoral -> 0

Axillary -> 1

Aortic, apical, carotid, caval, or subclavian -> 2

**Figure 3S.** Distribution of the TAVI-CT (transcatheter aortic valve implantation-computed tomography) score.

**Figure 4S.** Receiver-operator characteristic (ROC) curve for the association between TAVI-CT (transcatheter aortic valve implantation-computed tomography) score and risk of vascular complications. se=standard error.

.

**Figure 5S.** Receiver-operator characteristic (ROC) curve for the association between TAVI-CT (transcatheter aortic valve implantation-computed tomography) score and risk of vascular complications, excluding planned access. se=standard error.
